# Gut Microbiota Analysis in Postoperative Lynch Syndrome Patients

**DOI:** 10.3389/fmicb.2019.01746

**Published:** 2019-07-30

**Authors:** Giorgia Mori, Beatrice Silvia Orena, Ilenia Cultrera, Giulia Barbieri, Alessandra M. Albertini, Guglielmina Nadia Ranzani, Ileana Carnevali, Maria Grazia Tibiletti, Maria Rosalia Pasca

**Affiliations:** ^1^Department of Biology and Biotechnology “Lazzaro Spallanzani”, University of Pavia, Pavia, Italy; ^2^Research Center for the Study of Hereditary and Familial Tumors, University of Insubria, Varese, Italy; ^3^Department of Pathology, ASST-Sette Laghi, Varese, Italy

**Keywords:** Lynch syndrome, hereditary cancer predisposition, fecal microbiota, 16S sequencing, fecal biomarkers

## Abstract

Lynch syndrome (LS) is a dominantly inherited condition with incomplete penetrance, characterized by high predisposition to colorectal cancer (CRC), endometrial and ovarian cancers, as well as to other tumors. LS is associated with constitutive DNA mismatch repair (MMR) gene defects, and carriers of the same pathogenic variants can show great phenotypic heterogeneity in terms of cancer spectrum. In the last years, human gut microbiota got a foothold among risk factors responsible for the onset and evolution of sporadic CRC, but its possible involvement in the modulation of LS patients’ phenotype still needs to be investigated. In this pilot study, we performed 16S rRNA gene sequencing of bacterial DNA extracted from fecal samples of 10 postoperative LS female patients who had developed colonic lesions (L-CRC) or gynecological cancers (L-GC). Our preliminary data show no differences between microbial communities of L-CRC and L-GC patients, but they plant the seed of the possible existence of a fecal microbiota pattern associated with LS genetic background, with *Faecalibacterium prausnitzii*, *Parabacteroides distasonis*, *Ruminococcus bromii*, *Bacteroides plebeius*, *Bacteroides fragilis* and *Bacteroides uniformis* species being the most significantly over-represented in LS patients (comprising both L-CRC and L-GC groups) compared to healthy subjects.

## Introduction

Lynch syndrome (LS) is one of the most common hereditary cancer syndromes, conferring a high lifetime risk of colorectal cancer (CRC), endometrial cancer (EC), ovarian cancer (OC), as well as of a number of other neoplasms. LS accounts for 3% of CRC and for 2% of EC cases, with a recently estimated prevalence of 1:279 in the general population (Win et al., 2017). Although LS appears relatively common across different ethnic groups, founder mutations make it more frequent in some populations, including Icelanders, French Canadians, and individuals of Ashkenazi Jewish ancestry (Boland et al., 2018). Cancer predisposition is transmitted as an autosomal dominant condition associated with heterozygous germline alterations in DNA mismatch repair (MMR) genes (*MLH1*, *MSH2*, *MSH6*, *PMS2*). Tumors of LS patients show peculiar molecular and biological features including MMR-deficiency and accumulation of length variations in repetitive DNA sequences (referred as MSI for microsatellite instability), as well as high levels of tumor-infiltrating lymphocytes (Boland et al., 2018).

When LS is assessed on the basis of personal and/or familial history of cancer, *MSH2* and *MLH1* germline lesions are found in the great majority of patients (up to 87%), with *MSH6* and *PMS2* germline defects only accounting for a minority of cases (Boland et al., 2018). On the other hand, population-based epidemiological studies have recently shown that, not only LS is less rare and less penetrant than previously thought, but that *PMS2* and *MSH6* pathogenic variants are the most prevalent, thus suggesting a lower cancer risk for *PMS2* and *MSH6* pathogenic variants compared to *MLH1* and *MSH2* (Win et al., 2017). According to the Prospective LS Database^1^, carriers of different MMR pathogenic variants show gender- and age-related specific patterns of cancer risk and survival (Moller et al., 2018). In addition to gene-specific risk, cancer risk in LS can also be modulated by different types of mutations, by a diverse genetic background (because of both interindividual and interethnic variability), as well as by environmental factors. On the whole, LS patients show great phenotypic heterogeneity, including tumor spectrum and age of onset, even among family members sharing the same mutation (Scott et al., 2001).

The spreading of high resolution next-generation sequencing (NGS) technologies has begun to elucidate the complex etiological relationship between gut microbiome and CRC and is nowadays well accepted that patients with sporadic CRC are characterized by imbalances in the composition of their gut microbiota (Liang et al., 2017; Yu et al., 2017; Clegg et al., 2018; Hale et al., 2018). Indeed, gut microorganisms can contribute to CRC development by influencing host gene expression and metabolic regulation, as well as local and systemic immune and inflammatory responses (Huycke et al., 2001; Zhang et al., 2016). Moreover, different studies aimed at characterizing gut microbiota throughout different stages of colorectal carcinogenesis, highlighted significant changes during cancer progression (Feng et al., 2015; Nakatsu et al., 2015; Sze et al., 2017; Mori et al., 2018). Recently, Dejea et al. (2018) found an enrichment of both *Escherichia coli* and *Bacteroides fragilis* toxigenic bacterial species in the colonic mucosa of patients with familial adenomatous polyposis (FAP), a syndrome characterized by benign precursor lesions (colonic polyps) and high predisposition to CRC. In particular, these two species produced secreted oncotoxins (colibactin and *B. fragilis* toxin BFT), overexpressed in the colonic tissue of patients. These findings highlight a new link between early carcinogenesis of the colon and bacteria (Dejea et al., 2018).

With the main aim of characterizing the gut microbial population of patients with a LS-associated genetic background, we were specifically interested in investigating the gut microbiota overall composition, diversity, and taxonomic pattern abundance in postoperative LS patients with/without colorectal lesions or other type of cancers; is a diverse microbial community responsible for the modulation of the LS phenotype? Accordingly, by sequencing 16S rRNA gene with an NGS-based approach, we performed a pilot investigation of the fecal microbiota of healthy females and LS female patients who had developed either gynecological cancer only (GC: EC and/or OC) or colonic lesions, with/without additional tumors.

## Materials and Methods

### Patients and DNA Samples

Patients were recruited at the Varese Hospital (Ospedale di Circolo di Varese-ASST dei Sette Laghi) where cancer genetic counseling and genetic testing are offered to patients with MMR defective CRCs and/or GCs, according to international recommendations^2^. Following informed consent (protocol 002719, approved June 2015 by the Ethics Committee of Ospedale di Circolo Varese, Italy), 10 LS postoperative female patients known to be carrier of MMR pathogenic mutations (Class 4 and Class 5 according to the InSiGHT criteria) (Thompson et al., 2014), were enrolled for this study (mean age at sample collection: 58.6). Five LS patients had developed GCs only (EC/OC) and five had developed colonic lesions (in 4 cases: CRC; in one case: 2 dysplastic polyps) with/without additional tumors. In the latter cases, the colonic lesions were the last occurring clinical manifestation. Patients were from unrelated families, with the exception of T1 and T2 subjects who were sisters. Mean ages at cancer diagnosis were 55.8 and 53.6 for CRC and GC, respectively (Table 1). All patients had undergone cancer surgery before the onset of the study and none of them had received any cancer therapy for at least 2 years before fecal sampling, with the exception of patient T10 who received the last chemotherapy 4 months before sample collection. Eight healthy females, without family history of cancer, were used as controls. At sample collection, the mean age of the healthy subjects was 51.3, comparable to the mean age of LS patients. Both patients and healthy subjects were of Italian origin and living in Lombardia region.

**TABLE 1:** Clinical and genetic data of patients enrolled in the study.

**Sample name**	**Group**	**Mutated gene**	**Age at cancer diagnosis**		**Additional tumors (age)**	**Age at fecal sample collection**
			**CRC**	**GC**		
CT1	H	–				66
CT10	H	–				23
CT11	H	–				47
CT12	H	–				64
CT2	H	–				67
CT4	H	–				67
CT7	H	–				45
CT8	H	–				31
T3	L-CRC	*PMS2*	46			55
T5	L-CRC	*MSH2*	63		EC and OC (47)	66
					Rectum (60)	
					Breast (61)	
T7	L-CRC	*MLH1*	55		Epithelioma (54)	61
					EC (55)	
T9^a^	L-CRC	*MSH6*	54		OC (33)	54
					Kidney and EC (53)	
T13	L-CRC	*MSH6*	61		EC and OC (46)	62
T1	L-GC	*MSH6*		58		60
T2	L-GC	*MSH6*		52		55
T4	L-GC	*MSH6*		51		64
T10	L-GC	*MSH6*		47		48
T11	L-GC	*MSH6*		60		61

*H, healthy controls; L-CRC, LS patients with colonic lesions; L-GC, patients with gynecological cancer (EC/OC, endometrial and/or ovarian cancer). ^*a*^Patient T9 developed 2 dysplastic polyps.*

Stool specimens were collected by each participant using the Stool Collection and Preservation System (Norgen Biotek Corp.) provided to each participant by the Varese Hospital. The collected samples were sent to the Department of Biology and Biotechnology “Lazzaro Spallanzani” (University of Pavia, Italy) and stored less than 8 weeks at 4°C, until DNA extraction. All samples were processed in the same way. DNA was extracted from feces using QIAamp DNA Stool Handbook kit (Qiagen), according to manufacturer’s instructions; the lysis temperature indicated in the standard protocol of the QIAamp^®^ DNA Stool Kit was increased from 70 to 95°C in order to allow the retrieval of gram-positive bacteria and DNAs were stored at –20°C prior to amplification steps.

### Illumina Sequencing

Before sending samples for preparation of Illumina libraries and sequencing, PCR amplification of the 16S region, using 16S V4 amplification primers (515F–806R) pair indicated in Caporaso and collaborators (Caporaso et al., 2011), was performed to check for the presence of 16S in the extracted DNA. PCR water was used as a negative control for PCR amplification to check for the presence of possible environmental contaminations. Illumina DNA libraries and sequencing were performed at MR DNA (www.mrdnalab.com, Shallowater, TX, United States) on a MiSeq platform following the manufacturer’s guidelines. Sequencing outputs were raw sequence data information. The Fastq processor application on the website www.mrdnafreesoftware.com created the file formats expected by QIIME2 for downstream analysis.

### Bioinformatic Analysis

The obtained reads were pre-processed and analyzed with the QIIME2 pipeline^3^ for taxonomic composition, alpha diversity, and beta diversity analysis.

The QIIME2 plugin “qiime taxa collapse” was used to collapse OTUs with the same taxonomic assignment. In being collapsed, OTUs frequencies were summed.

Alpha diversity was measured using three different indices (Shannon, Fisher, and Coverage) and compared between the different groups of samples using Kruskal–Wallis test (“qiime diversity alpha-group-significance” QIIME2 plugin). *P*-values were corrected with a Benjamini and Hochberg correction method and false discovery rate (FDR) correction was applied as multiple comparisons method. FDR < 0.05 was considered as statistically significant. The *R* phyloseq package was used to import and graphically display the resulting alpha diversity measures (McMurdie and Holmes, 2013).

Principal coordinate analysis (PCoA) ordination was performed based on Weighted and Unweighted UniFrac distances using QIIME2 and Phyloseq package (McMurdie and Holmes, 2013). Data separation in the PCoA was tested using the ADONIS permutation-based statistical test in vegan-R, and *p*-values were generated based on 999 permutations (“qiime diversity adonis” QIIME2 plugin).

The univariate DESeq2 method was used to identify differentially abundant OTUs (Love et al., 2014; Thorsen et al., 2016). DESeq2 is based on the negative binomial Wald test; statistically significant results are considered *p*-values < 0.05 and were adjusted for FDR using the method described by Benjamini and Hochberg (1995).

## Results

**FIGURE 1:**
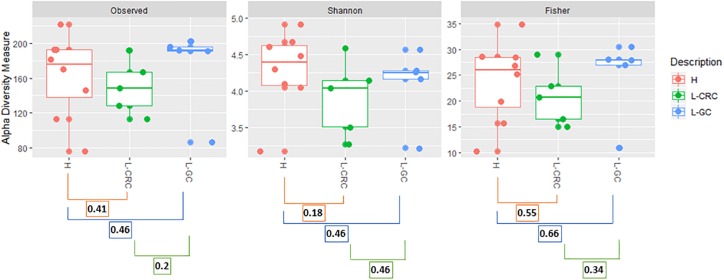
Observed_OTUs, Shannon and Fisher alpha diversity indices. Kruskal–Wallis test was used to compare alpha diversity between healthy (H) subjects and LS patients with CRC (L-CRC) or gynecological cancer (L-GC). *p*-values for each group comparison are reported. *p*-values were corrected with a Benjamini and Hochberg correction method and false discovery rate (FDR) correction was applied as multiple comparisons method.

A total of 1264 operational taxonomic units (OTUs) were delineated at a 99% similarity level, with sequence counts ranging from 16997 to 38119 (Supplementary Table S1). Shannon, Fisher, and Coverage alpha diversity indices showed comparable microbial richness among the three groups of subjects, i.e., healthy females (H) and postoperative LS patients who had developed either GCs only (L-GC) or colonic lesions with/without additional tumors (L-CRC) (Figure 1).

The taxonomic composition identified in our healthy subjects and LS patients (L-GC and L-CRC) reflects a microbial community typical of the intestinal microbiota. In the three groups, the four predominant phyla were Bacteroidetes, Firmicutes, Actinobacteria, and Proteobacteria. Among the most prevalent families we found Lachnospiraceae, Bifidobacteriaceae, Bacteroidaceae, Ruminococcaceae, and Rikenellaceae (Table 2). A total of 118 different taxa were identified in all samples at genus level, with the most representative being reported in Supplementary Figure S1. No significant differences were detected between H, L-GC, and L-CRC subjects.

**TABLE 2:** Abundance of the most dominant phyla and families detected in all patients and controls (a threshold greater than 0.5% was applied).

	**Study groups**		
	**H**	**L-GC**	**L-CRC**
	**Mean (%) ± SD**	**Mean (%) ± SD**	**Mean (%) ± SD**
**Phylum**			
Bacteroidetes	37.9 ± 0.09	23.51 ± 0.04	19.46 ± 0.07
Firmicutes	55.7 ± 0.09	61 ± 0.16	56.68 ± 0.16
Actinobacteria	1.57 ± 0.006	4.96 ± 0.04	11.74 ± 0.16
Proteobacteria	3.05 ± 0.01	7.93 ± 0.11	2.75 ± 0.01

**Family**			
Lachnospiraceae	23.14 ± 0.06	13.72 ± 0.06	19.92 ± 0.06
Bifidobacteriaceae	0.71 ± 0.005	4.33 ± 0.04	11.02 ± 0.17
Bacteroidaceae	23.6 ± 0.14	14.32 ± 0.06	13.52 ± 0.07
Ruminococcaceae	24.6 ± 0.08	31.28 ± 0.12	27.74 ± 0.18
Rikenellaceae	9 ± 0.09	5.31 ± 0.08	3.83 ± 0.02

*Mean values are expressed in percentage.*

**FIGURE 2:**
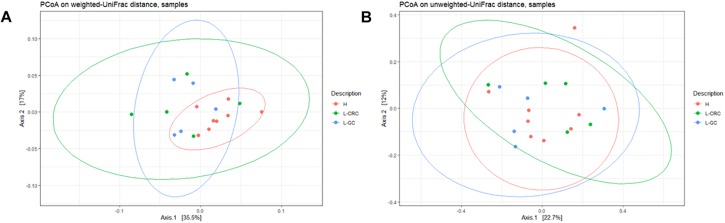
**(A)** PCoA on weighted UniFrac distance showing clusterization of H compared to L-CRC and L-GC groups (*p*-value = 0.013). **(B)** PCoA on unweighted UniFrac distance showing the absence of clusterization of H, L-CRC and L-GC groups (*p*-value = 0.37). The analysis were generated by the “qiime diversity adonis” QIIME2 plugin and the *p*-values were calculated using the ADONIS permutation-based statistical test.

Unweighted and weighted UniFrac distances were performed to compare the overall structure of the gut microbiota of all samples based on the OTUs relative abundance. Weighted UniFrac analysis showed a statistically significant compositional difference between healthy subjects and L-GC or L-CRC patients (*p*-value = 0.013) (Figure 2A), whilst no significant differences exist between L-GC and L-CRC patients (*p*-value = 0.84). Unweighted UniFrac distance revealed the absence of significant compositional difference between healthy subjects and L-GC or L-CRC patients (*p*-value = 0.37), as well as between L-GC and L-CRC patients (*p*-value = 0.73) (Figure 2B).

Since we did not detect significant differences in microbial composition among L-GC and L-CRC groups, we analyzed L-GC and L-CRC as a single LS group of patients, in order to investigate which were those OTUs quantitatively different in abundance between the H subjects and LS patients, responsible for the microbial community dissimilarity highlighted by the weighted UniFrac distance.

**FIGURE 3:**
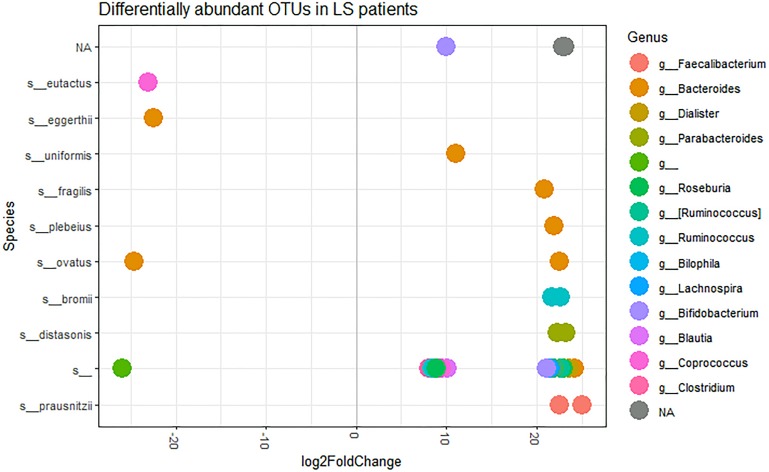
log2FoldChange OTUs representation, at the species level, in LS patients, performed using DeSeq2 with an official extension within the phyloseq package. Each dot represents a single OTU. Sequencing taxonomic analysis revealed 41 defined species and 113 unclassified species. Here, only those species showing significant differences in abundance between the H and LS groups are shown.

In order to identify possible differences in OTUs abundance between LS patients and controls, we used DESeq2 package (Love et al., 2014). Based on the log fold change of the mean normalized read counts, 32 OTUs proved to be significantly over-represented and 3 OTUs significantly under-represented in LS patients compared to controls (Supplementary Table S2 and Figure 3). The under-represented OTUs included: one undefined species of the Rikenellaceae family, *Coprococcus eutactus*, and *Bacteroides eggerthii* species. Among the over-represented species, we found OTUs belonging to: *Faecalibacterium prausnitzii* (2 OTUs), *Parabacteroides distasonis* (2 OTUs), *Ruminococcus bromii* (2 OTUs), *Bacteroides plebeius*, *B. fragili*s and *Bacteroides uniformis*. The remaining 23 OTUs were from unclassified species belonging to *Bacteroides*, *Dialister*, *Roseburia*, *Ruminococcus*, *Bilophila*, *Lachnospira*, *Bifidobacterium*, *Blautia*, *Coprococcus* and *Clostridium* genera. The OTUs belonging to unclassified genera were from Ruminococcaceae and Lachnospiraceae families.

## Discussion

In this pilot study, we analyzed the gut microbiota composition of LS female patients showing different clinical phenotypes: L-GC patients had developed EC and/or OC only, while L-CRC patients had developed colonic lesions, with/without other neoplasias. In spite of differences in tumor types, the two subgroups did not show significant changes in the overall structure of their gut microbiota. However, we observed a statistically significant compositional difference in the weighted UniFrac analysis between our healthy subjects compared to L-CRC and L-GC (Figure 2A), compared to the unweighted UniFrac analysis (Figure 2B). Therefore, OTUs relative abundances were responsible for a different gut microbiota composition among these groups.

Many of the OTUs we have identified with our analysis did not have a species-level classification, meaning that OTUs belonging to the same genus, and are in reality different species, will all be collapsed into the same species-level feature. We then decided to avoid collapsing OTUs and, since no differences in microbial composition were detected between L-CRC and L-GC groups, we considered these two groups as a single LS group, in order to compare the relative abundances of the single OTUs identified within the LS group against the relative abundances of the single OTUs identified in the healthy subjects.

In this way, we could detect a significant difference in some OTUs abundances between LS postoperative patients and healthy female controls.

These OTUs, identified as *F. prausnitzii*, *P. distasonis*, *R. bromii*, *B. plebeius*, *B. fragilis*, and *B. uniformis*, were over-represented in LS patients and already reported in literature as being linked either to CRC or to healthy status (Hibberd et al., 2017; Mangifesta et al., 2018). In particular, *B. uniformis* and *B. plebeius* extract energy from recalcitrant polysaccharides using carbohydrate active enzymes, adding new catabolic functions to the human gut microbiome (Hehemann et al., 2012). Recently, *P. distasonis* was demonstrated to generate succinate and secondary bile acids in the gut, suggesting that it could be used as a probiotic (Wang et al., 2019). The 70% of the energy achieved by intestinal epithelial cells originates from butyrate produced by gut bacteria belonging to *Ruminococcus* and *Faecalibacterium* genera (Hibberd et al., 2017; LeBlanc et al., 2017; Mangifesta et al., 2018). *F. prausnitzii* is one of the most abundant butyrate producing species in the gut. Its beneficial effects have also been attributed to the production of salicylic acid, another anti-inflammatory metabolite (Ferreira-Halder et al., 2017; Martin et al., 2018). The protective action of this species was further demonstrated by Wei and collaborators (Wei et al., 2016), finding a higher abundance of *F. prausnitzii* species in survival group of CRC patients. Similarly, a recent study (Sze et al., 2017) showed that the fecal microbiota composition of sporadic CRC patients changed after cancer treatment, shifting toward a normal healthy microbiota.

Of relevance, the patients enrolled in this pilot study received the last surgical/pharmacological treatment at least 1 year before feces sampling. We can speculate that this long disease- and treatment-free interval allowed LS patients’ microbiota to shift closer to its original condition (i.e., as before cancer development). If so, our preliminary observations suggest a microbiota pattern differing between normal subjects and pre-symptomatic LS subjects.

In a recent communication, Lu et al. (2017) reported that while no differences in the gut microbiota composition were detectable between LS cancer patients and asymptomatic mutation carriers, LS subjects were significantly different from normal subjects. In particular, in agreement with our observations, *B. fragili*s and *P. distasonis* species proved to be over-represented in LS cases.

On the whole, these observations and our preliminary data point to a microbiota composition associated with LS genetic background. Although we did not detect differences between L-CRC and L-GC, these could be because of the limited number of samples, or even because of the different type of genetic mutation.

All of the recruited L-GC patients are characterized by a *MSH6* gene mutation, whereas L-CRC patients harbor mutations in either *MSH6*, *MLH1*, *PMS2* or *MSH2* gene. Although referred to a very small cohort, this reflects previous findings on predominant *MSH6* gene mutations when LS screening is performed on EC patients (Hampel et al., 2006). Because of the limited number of samples, we could not investigate whether a different LS-associated genetic background is characterized by a different gut microbial population. The recruitment of a larger cohort of patients could be of help in determining this aspect. In addition, fecal sample collection before patient’s surgeries, as well as recruitment of LS mutation carriers without a developed cancer, should be performed in order to identify gut microbiota species that can be used as potential biomarkers for the detection of CRC development in LS patients.

The application of metagenomic analysis should also be considered for better characterize bacterial species potentially involved in LS-associated pathophysiology of CRC.

To date, no other studies are present in literature investigating the gut microbial community of LS patients. The preliminary data we reported in this paper represent a starting point for the investigation of bacterial communities that could be involved in CRC development in LS patients genetically predisposed to cancer. Moreover, screening for LS-related cancers is offered to individuals at risk for LS, who have either not undergone genetic evaluation or have uncertain genetic test results. The characterization of a gut microbiota associated with an increased risk of developing CRC in these patients could be of important impact for prevention purposes.

## Data Availability

The raw data supporting the conclusions of this manuscript have been deposited into NCBI’s Sequence Read Archive (SRA) with the Accession Number PRJNA532948 (https://www.ncbi.nlm.nih.gov/sra/PRJNA532948).

## Ethics Statement

This study was carried out in accordance with the recommendations of the Ethics Committee of Ospedale di Circolo Varese, Italy, with written informed consent from all subjects. All subjects gave written informed consent in accordance with the Declaration of Helsinki. The protocol 002719 was approved by the Ethics Committee of Ospedale di Circolo Varese, Italy in June 2015.

## Author Contributions

GM, AA, GR, MT, and MP planned the experiments. GM, BO, and GB performed the experiments. GM and ICu performed the bioinformatic and statistical analysis. GR, MT, and ICa characterized the Lynch syndrome patients and provided biological samples. All authors contributed to the data interpretation and manuscript preparation, and approved the final version of the manuscript.

## Conflict of Interest Statement

The authors declare that the research was conducted in the absence of any commercial or financial relationships that could be construed as a potential conflict of interest.

## Footnotes

1 http://lscarisk.org/

2 https://www.insight-group.org/syndromes/lynch-syndrome/

3 https://qiime2.org/
