# Supplementary material for: Gut Microbiota Analysis in Postoperative Lynch Syndrome Patients
Source: Front Microbiol. 2019 Jul 30;10:1746. doi: 10.3389/fmicb.2019.01746 (PMC6682596; doi:10.3389/fmicb.2019.01746)
Supplement: Supplementary file 1 [file Data_Sheet_1.PDF]

Supplementary Material

Legends to Supplementary Figures

Supplementary Figure 1. Abundance of the main genera detected in each sample. In total, 118 OTUs were detected at genus level in the 18 samples. The most prevalent genera are listed in figure, alongside with the less representative ones (indicated as "g__").
