# Supplementary figures and images for: Gut Microbiota Analysis in Postoperative Lynch Syndrome Patients
Source: Front Microbiol. 2019 Jul 30;10:1746. doi: 10.3389/fmicb.2019.01746 (PMC6682596; doi:10.3389/fmicb.2019.01746)

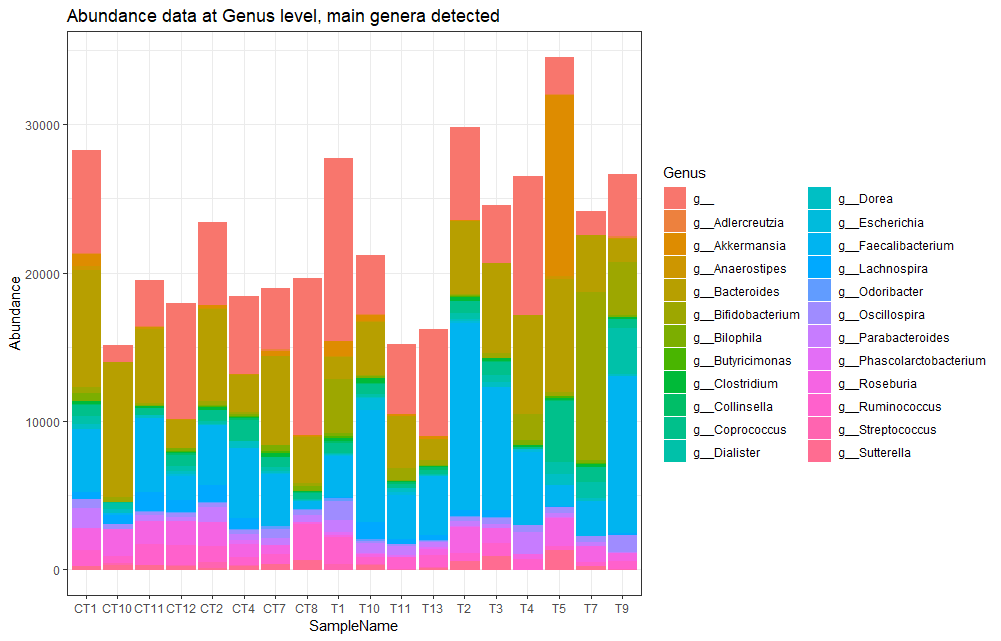

Supplement: Supplementary file 2 [file Image_1.TIF]
