# Supplementary material for: Gut Microbiota Analysis in Postoperative Lynch Syndrome Patients
Source: Front Microbiol. 2019 Jul 30;10:1746. doi: 10.3389/fmicb.2019.01746 (PMC6682596; doi:10.3389/fmicb.2019.01746)
Supplement: Supplementary file 3 [file Table_1.DOCX]

| **Sample name** | **Total sequence count** | **Filtered reads** | **Denoised reads** | **Merged reads** | **Non-chimeric reads** |
| --- | --- | --- | --- | --- | --- |
|  |  |  |  |  |  |
| CT1 | 62541 | 52600 | 52600 | 47957 | 30968 |
| CT10 | 54156 | 47090 | 47090 | 41416 | 16997 |
| CT11 | 52713 | 46343 | 46343 | 41889 | 20737 |
| CT12 | 44186 | 37853 | 37853 | 34279 | 20170 |
| CT2 | 60401 | 51236 | 51236 | 47304 | 25000 |
| CT4 | 58966 | 50844 | 50844 | 46912 | 24662 |
| CT7 | 57315 | 49274 | 49274 | 41793 | 21637 |
| CT8 | 61278 | 52674 | 52674 | 46021 | 22768 |
| T3 | 66488 | 54350 | 54350 | 49824 | 26460 |
| T5 | 82196 | 67282 | 67282 | 61098 | 38119 |
| T7 | 73946 | 60701 | 60701 | 56564 | 27899 |
| T9 | 72001 | 60942 | 60942 | 56108 | 33320 |
| T13 | 57420 | 48992 | 48992 | 44508 | 21794 |
| T1 | 91156 | 74801 | 74801 | 67862 | 30627 |
| T2 | 95305 | 78659 | 78659 | 70039 | 32564 |
| T4 | 75582 | 61188 | 61188 | 57421 | 28628 |
| T10 | 53588 | 46232 | 46232 | 41833 | 22891 |
| T11 | 62523 | 54108 | 54108 | 50443 | 26865 |

**Supplementary Table 1.** Sequencing details of each sample from the “qiime dada2 denoise-single” QIIME2 plugin.
