# Supplementary material for: Gut Microbiota Analysis in Postoperative Lynch Syndrome Patients
Source: Front Microbiol. 2019 Jul 30;10:1746. doi: 10.3389/fmicb.2019.01746 (PMC6682596; doi:10.3389/fmicb.2019.01746)
Supplement: Supplementary file 4 [file Table_2.DOCX]

**Supplementary Table 2.** DESeq2 output data. The *p* values (pvalue column) are calculated with the Wald test. The adjusted *p* values (padj column) are calculated with Benjamini & Hochberg adjustment method. Log2FoldChange values are reported for each OTU.
